# Draft genome sequence of *Bacillus cereus* SBI_SS06_AR_BD: a bacterium producing both alpha-amylase and lipase

**DOI:** 10.1128/mra.00267-25

**Published:** 2025-10-08

**Authors:** Asfia Islam Raida, Md. Safayat Sahib, Raiyan Ul Hakim, Jisan Raihan, Md. Abu Tayeb, Md. Fakruddin, Spencer Mark Mondol, S. M. Bakhtiar UL Islam

**Affiliations:** 1 Department of Biochemistry and Microbiology, School of Health & Life Sciences, North South University 54495, Dhaka, Bangladesh; 2 Department of Microbiology, University of Dhaka 95324, Dhaka, Bangladesh; University of Maryland School of Medicine, Baltimore, Maryland, USA

**Keywords:** alpha-amylase, *Bacillus cereus*, Bangladesh, BV-BRC, lipase

## Abstract

*Bacillus cereus* is a Gram-positive, rod-shaped, spore-forming soil bacterium with a natural ability to produce industrially valuable enzymes. Here, we report the 5,132,550 bp genome (35.15% G + C) of *Bacillus cereus* SBI_SS06_AR_BD, containing a total of 5,220 coding sequences, 27 tRNA genes, and 3 rRNA genes—an alpha-amylase and lipase-producing strain from Bangladesh.

## ANNOUNCEMENT

*Bacillus* spp. are Gram-positive, rod-shaped, spore-forming soil bacteria, some of which are foodborne pathogens, while others hold significant biotechnological potential due to their potential ability to produce industrially relevant enzymes (1, 2). Notably, *Bacillus cereus* produces stable extracellular alpha-amylase and lipase, which are enzymes valuable for industrial use (3). These enzymes break down starch and lipids and are widely applied in biotechnology (4). The resilience of *Bacillus cereus* to diverse environmental conditions, such as temperatures, pH levels, and alkaline conditions, makes it well-suited for large-scale fermentation (5). Advances in genomics and metabolic engineering offer promising avenues to enhance enzyme yield and unlock its full industrial potential (6).

*Bacillus cereus* SBI_SS06_AR_BD was isolated from BCIC industrial effluent surrounding area of BSCIC Tannery industrial state (Latitude: 23°78′22.33623733905″E, 90°24′27,8869413872″N), Hemayetpur, Savar, Bangladesh, in 2023. In brief, soil samples were collected in a zip lock bag and transported to the microbiology laboratory of North South University. After 10-fold serial dilutions of the soil sample, pure cultures were isolated in nutrient agar media for primary enzyme activity screening in starch agar media. To isolate heat-resistant bacterial strains, the diluted samples were then heat-treated at 80°C for 1 h. After presumptive identification by Gram staining, endospore staining, biochemical tests, growth on mannitol egg yolk polymyxin (MYP) selective media, and primary enzyme activity confirmation in starch agar media, this isolate was selected for whole genome sequencing for genome analysis to understand the industrial applicability.

Prior to DNA isolation, the strain *B. cereus* SBI_SS06_AR_BD was grown in nutrient agar media at 37°C overnight and sent for whole genome sequencing at the Genome Sequencing Laboratory, BCSIR, Dhaka, Bangladesh. Genomic DNA (gDNA) was isolated using the gDNA extraction by Qiagen DNeasy Blood & Tissue Kit (Hilden, Germany), following the manufacturer’s protocol. For genome sequencing, libraries were constructed using the Nextera DNA Flex Library Preparation Kit (Illumina, San Diego, USA) and paired-end raw reads (2 × 150 bp) were generated using the Illumina NextSeq 550 platform.

Genome quality assessment and assembly were conducted using workflows available through the Bacterial and Viral Bioinformatics Resource Center (BV-BRC). Trim Galore version: 0.6.5dev was used for quality control (7) and trimming of paired-end reads (8), and *de novo* assemblies was performed using Unicycler v0.4.8 (9) as set in the BV-BRC pipeline version 3.46.3 (10). The genome size was determined to be 5,132,550 bp with an approximate genome coverage of 287× and an average G + C content of 35.15%. This sequence exhibited good quality with coarse consistency, fine consistency, contamination, and checkM completeness values 99.7%, 98.7%, 0%, and 100%, respectively. Genome assembly resulted in 39 contigs (>300 bp), with an N_50_ of 241,419 bp and L_50_ of 7. Genome annotation for *B. cereus* SBI_SS06_AR_BD was performed using the NCBI Prokaryotic Genome Annotation Pipeline (PGAP) version 6.10 (11) using default parameter. This draft genome has a total of 5,220 coding sequences, 27 tRNA, and 3 rRNA genes. Genomic analysis showed the presence of one alpha-amylase (1,356 bp) and multiple lipase (723, 834, 936, and 1,602 bp) genes.

## Data Availability

The data described in this report are publicly accessible through NCBI BioProject PRJNA1234558 and BioSample SAMN47301816. The draft genome sequences have been deposited in GenBank under SRA accession number SRR32653847, with a release date of March 20, 2025. This Whole Genome Shotgun project has been deposited at DDBJ/ENA/GenBank under the accession JBQSFC000000000. The version describedin this paper is version JBQSFC010000000.1.
